# Fixel-based analysis identifies selective vulnerability of non-dominant fiber populations in aging and mild cognitive impairment

**DOI:** 10.1007/s00429-026-03224-3

**Published:** 2026-11-14

**Authors:** Aditi Sathe, Ankita Chatterjee, Niranjana Shashikumar, Kimberly R. Pechman, Kurt Schilling, Bennett A. Landman, Leslie S. Gaynor, Corey J. Bolton, Panpan Zhang, Timothy J. Hohman, Angela L. Jefferson, Andrew R. Bender, Derek B. Archer

**Affiliations:** 1 Vanderbilt Memory and Alzheimer’s Center, Vanderbilt Health, 3319 West End Avenue, 8th Floor, Nashville, TN 37203 USA; 2 Vanderbilt Genetics Institute, Vanderbilt Health, Nashville, TN USA; 3 Vanderbilt University Institute of Imaging Science, Vanderbilt Health, Nashville, TN USA; 4 Department of Radiology and Radiological Sciences, Vanderbilt Health, Nashville, TN USA; 5 Department of Electrical and Computer Engineering, Vanderbilt University, Nashville, TN USA; 6 Department of Medicine, Vanderbilt Health, Nashville, TN USA; 7 Department of Biostatistics, Vanderbilt Health, Nashville, TN USA; 8 Cleveland Clinic, Lou Ruvo Center for Brain Health, Las Vegas, NV USA; 9 Vanderbilt Alzheimer’s Disease Research Center, Nashville, TN USA

**Keywords:** Fixel-based analysis, Diffusion MRI, Alzheimer’s disease, Neuroimaging, Cognition, White matter

## Abstract

Fixel-based analysis (FBA) resolves fiber-specific white matter properties within crossing-fiber regions, where conventional diffusion tensor imaging is limited. We applied single-shell three-tissue constrained spherical deconvolution to diffusion MRI from 295 older adults in the Vanderbilt Memory and Aging Project cohort, quantifying fiber density (FD), fiber-bundle cross-section (FC), and their composite (FDC) across the primary (N1), secondary (N2), and tertiary (N3) fiber populations within each white matter voxel. White matter was stratified into crossing-fiber complexity strata defined by the number of distinct bundles per voxel, and covariate-adjusted models examined associations with age, cognitive status, and cognition. FC declined with age most steeply in the non-dominant fiber populations. In these populations, FC was lower in participants with mild cognitive impairment than in cognitively unimpaired participants, and higher FC was associated with better executive function. The age-related decline in non-dominant FC did not vary across crossing-fiber complexity, indicating that fiber-population identity, rather than local architecture, organizes age-related vulnerability. FD and FDC showed age associations that became more positive as complexity increased, with the N1 FD association reversing from negative in the least complex regions to positive in the most complex. This behavior is consistent with how density is estimated in multi-fiber voxels rather than with fiber loss, identifying FC as the more interpretable metric in crossing-fiber regions. These findings identify fiber cross-section in the non-dominant fiber populations as a sensitive marker of white matter aging and cognitive status and provide a framework for fiber-specific analysis in existing single-shell cohorts.

**Supplementary Information:**

The online version contains supplementary material available at https://doi.org/10.1007/s00429-026-03224-3.

## Introduction

Age-related white matter alterations in the brain have consistently been linked to changes in cognitive function in both healthy and impaired aging (Bendlin et al., 2010; Ferro and Madureira, 2002; Madden et al., 2009). White matter volume (Liu et al., 2017) and tract microstructure (Benitez et al., 2018) have been noted to decline with age, particularly through mechanisms that result in myelin damage (Gong et al., 2023) and axonal degeneration (Salvadores et al., 2017). Characterizing the spatial and anatomical specificity of these white matter alterations is essential to understanding the biological substrates of cognitive aging (Bartzokis et al., 2004).

Diffusion tensor imaging (DTI) has served as the primary non-invasive modality for assessing white matter microstructure (Aung et al., 2013; Kennedy and Raz, 2009), using scalar metrics such as fractional anisotropy (FA) (Voineskos et al., 2012) and mean diffusivity (MD) (Cox et al., 2016). Accumulating evidence has implicated age-related axonal degeneration (Salvadores et al., 2017) and myelin damage (Nasrabady et al., 2018) as critical contributors to the pathophysiological cascade of Alzheimer’s disease (AD) (Bartzokis et al., 2003; Papuć and Rejdak, 2018), primarily through the selective vulnerability and subsequent degeneration of late-myelinating neurons (Braak et al., 2000). DTI studies consistently demonstrate that the greatest age-related reductions in FA occur within later-developing, less myelinated association white matter tracts (Bender and Raz, 2015; Kochunov et al., 2009), whereas earlier-developing heavily myelinated primary motor and sensory pathways exhibit comparatively minimal changes (Bender, Völkle, et al., 2016; Garnier-Crussard et al., 2022; Peter et al., 2025). Notably, microstructural alterations of long-range association fibers, including the superior longitudinal fasciculus (SLF), inferior longitudinal fasciculus (ILF), uncinate fasciculus (UF), and cingulum bundle (Huang et al., 2012; Lee et al., 2015; Mayo et al., 2017; Stone et al., 2021), emerge as some of the most robust and reproducible neuroimaging correlates of cognitive decline across the AD continuum. Recent work from our group using data from the Vanderbilt Memory & Aging Project (VMAP) has demonstrated that these tracts are strongly associated with cognitive decline. For example, recent reports from VMAP show that the ILF and cingulum bundle are associated with both cognitive impairment and decline; moreover, the microstructure of these tracts contributes independently to cognitive decline beyond that of hippocampal volume (Archer et al., 2020). Furthermore, large-scale work from our group spanning nine cohorts (*n* = 4,467) identified white matter free water, particularly within limbic tracts such as the cingulum and fornix, as the microstructural measure most strongly associated with both cognitive performance and decline (Peter et al., 2025).

A fundamental limitation of the DTI single-tensor model is its inability to resolve complex fiber configurations within a voxel (Figley et al., 2022; Landman et al., 2012). This is a critical shortcoming, as regions including crossing, branching, and converging fibers are estimated to constitute 60–90% of the brain white matter (Jeurissen et al., 2012). Importantly, the white matter regions with the greatest vulnerability to disease-related alterations in cognitive impairment and AD, such as the cingulum bundle and SLF, exhibit a high degree of complexity in fiber structure and orientation (Douaud et al., 2011). Thus, to detect early microstructural changes that may be obscured by methods aggregating signals from multiple fiber populations within a voxel, it is crucial to analyze distinct fiber populations separately. This approach enables the independent assessment of potentially affected and unaffected pathways within regions of complex white matter architecture (Bender et al., 2025; Kelley et al., 2021; Sánchez et al., 2020).

Fixel-based analysis (FBA) (Raffelt et al., 2017) is an alternative diffusion MRI (dMRI) modeling and analysis framework designed to resolve individual fiber populations or “fixels” with different spatial orientations within a voxel. FBA allows for the estimation of specific microstructural properties, including fiber density (FD), fiber-bundle cross section (FC), and their composite measure fiber density modulated by cross section (FDC), which captures both microstructural and macrostructural white matter characteristics independently for multiple intravoxel fiber populations (Dhollander et al., 2021). Compared to traditional methods, FBA offers greater anatomical specificity (Raffelt et al., 2015) and recent applications of FBA show more nuanced patterns of white matter degeneration in a wide range of neurological disorders than traditional tensor modeling (Finkelstein et al., 2021; Haykal et al., 2019; Kirkovski et al., 2020; Oh et al., 2021).

Recent evidence demonstrates that fixel-based metrics differ significantly across diagnostic groups along the AD continuum (Mito et al., 2018). These effects are particularly prevalent within the fornix (Bhattarai et al., 2023) and SLF (Billaud et al., 2024), suggesting their potential use as markers of AD-relevant endophenotypes. Specifically, FC in the parahippocampal cingulum exhibits negative correlations with tau-PET uptake (Ahmadi et al., 2024), and FD and FDC in the fornix and medial temporal lobe fibers exhibit positive correlations with memory performance (Billaud et al., 2024). Age-related fixel associations likewise vary substantially by region, with the strongest effects reported in the fornix and in striatal and thalamic pathways (Tinney et al., 2024), consistent with the heterogeneous distribution of microstructural change in aging and early-stage AD (Wen et al., 2019).

Prior applications of FBA have largely examined fixel metrics within tracts or regions of interest (ROIs), without directly comparing the distinct fiber populations that cross within a single voxel. The present study extends this work by examining fixel metrics separately across primary (N1), secondary (N2), and tertiary (N3) fiber populations throughout the brain. These populations are defined by the rank of each fixel’s fiber density within its voxel rather than by anatomical identity, so N1, N2, and N3 do not correspond to fixed named tracts and their composition varies across the brain. Assigning each population to named pathways would require tractography and is beyond the scope of the present analysis. We evaluated associations of FD, FC, and FDC with age, cognitive status (cognitively unimpaired, CU, versus mild cognitive impairment, MCI), and baseline and longitudinal cognitive performance. To examine whether fiber architecture modulates white matter microstructure, we further stratified the white matter into four crossing-fiber complexity strata defined by the number of distinct bundles traversing each voxel. To our knowledge, this is the first study to stratify the white matter by in vivo crossing-fiber complexity in order to test whether anatomical fiber complexity modulates age-related and disease-related differences across multiple fiber populations.

We hypothesized that the secondary and tertiary fiber populations (N2 and N3) would show stronger associations with age, cognitive status, and cognitive performance than the primary population (N1). This expectation follows from the observation that non-dominant populations more often comprise later-developing, thinly myelinated association fibers, which mature last and are more susceptible to age-related and disease-related change (Bender and Raz, 2015; Kochunov et al., 2009). Because FD and FC are estimated differently, with FD depending on how the within-voxel signal is apportioned among resolved populations and FC derived from the registration warp (Dhollander et al., 2021; Raffelt et al., 2017), we did not expect the two metrics to behave equivalently across fiber populations. We further asked whether crossing-fiber complexity modulates these associations. Given the role of crossing-fiber regions in maintaining large-scale network efficiency (Hua et al., 2009; Wiegell et al., 2000), we considered the possibility that the most complex regions would show more pronounced differences in the non-dominant fiber populations, and we tested this directly by stratifying the white matter by crossing-fiber complexity.

## Methods

### Study cohort

The Vanderbilt Memory & Aging Project (VMAP) (Jefferson et al., 2016) is a longitudinal observational study of vascular health and brain aging. All VMAP participants are aged 60 + years and inclusion is limited to those considered cognitively unimpaired (CU) or have mild cognitive impairment (MCI). MCI determinations were based upon National Institute on Aging/Alzheimer’s Association Workgroup core clinical criteria (Albert et al., 2011). VMAP participants (n_participants_=295, n_observations_=1193, age at baseline: 72.9 ± 7.4 years, 44% MCI at baseline) underwent longitudinal neuropsychological assessment and brain MRI over a maximum of seven time points including baseline (*n* = 295), 18-month (*n* = 222), 3-year (*n* = 219), 5-year (*n* = 187), 7-year (*n* = 148), 9-year (*n* = 97), and 11-year (*n* = 25) visits. The mean follow-up period was 4.6 ± 2.7 years. In the present study, fixel metrics were derived from the baseline diffusion MRI only, whereas cognitive performance was analyzed across all available visits. At study entry, participants completed a comprehensive clinical evaluation, including fasting blood draw, physical examination, clinical interview with medication review, echocardiogram, and brain MRI (Moore et al., 2021). As part of the screening assessment, participants were excluded for a cognitive diagnosis of dementia, MRI contraindication, history of neurological disease (e.g., multiple sclerosis, stroke), heart failure, major psychiatric illness, head injury with loss of consciousness > 5 min, or a systemic or terminal illness affecting follow-up participation. Informed consent was provided by all participants, and the Vanderbilt Institutional Review Board approved the protocol. Several demographic and clinical covariates were required for inclusion in the present study, including age, sex, educational attainment, race/ethnicity, apolipoprotein E (*APOE*) genotype (ε2, ε3, ε4), Framingham Stroke Risk Profile (FSRP; excluding points for age), and intracranial volume (ICV). Race and ethnicity were self-reported.

### Neuropsychological assessment

Participants completed a comprehensive neuropsychological protocol assessing language, information processing speed, executive function, visuospatial skills, and episodic learning and memory. Psychometrically sound composites for memory and executive function were created using a previously described method (Kresge et al., 2018). Briefly, the memory composite compiled scores from the California Verbal Learning Test-Second Edition (CVLT-II) Total Learning, Interference Condition, Long Delay Free Recall, and Recognition components (Delis et al., 2000), together with identical components of the Biber Figure Learning Test (Glosser et al., 2002). For the executive functioning composite, the Delis-Kaplan Executive Function System (DKEFS) Tower Test, DKEFS Letter-Number Switching, DKEFS Color-Word Inhibition (Delis et al., 2001), and Letter Fluency (FAS) (Patterson, 2011) test scores were evaluated. The Animal Naming Test (Campagna et al., 2017) and Boston Naming Test (Kaplan et al., 1983) scores were used to quantify language performance, and WAIS-IV Coding (Wechsler, 2008), and DKEFS Number Sequencing (Delis et al., 2001) scores assessed information processing speed. The Hooper Visual Organization Test (Buttaro, 2011) was used to evaluate visuospatial skills.

### Diffusion MRI acquisition and preprocessing

Participants underwent MRI scans at the Vanderbilt University Institute of Imaging Science on a 3 T Philips Achieva system (Best, The Netherlands). Imaging parameters are detailed in prior reports of this cohort (Jefferson et al., 2016; Moore et al., 2018). Briefly, we acquired dMRI images with a spin-echo echo-planar sequence, repetition time = 10 s, echo time = 60 ms, spatial resolution = 2 mm isotropic, and b-values of 1000 s/mm^2^, collected along 32 diffusion gradient encoding directions, and one non-diffusion weighted (B0) image. Comprehensive dMRI preprocessing and QA were conducted using the *PreQual* pipeline (Cai et al., 2020), which includes denoising, eddy current correction, and motion correction. As no reverse phase-encoded (RPE) image was acquired during data collection, we used Synb0-DisCo (Schilling et al., 2019) to generate a synthetic reverse phase-encoded b0 image for subsequent preprocessing. Although diffusion MRI was acquired at multiple visits in this cohort, only the baseline scan was analyzed here, and all fixel metrics are therefore cross-sectional.

### Quantifying fixel metrics

FBA metrics were computed using the MRtrix3 software suite (Tournier et al., 2019), following recommended procedures for single-shell data. For each participant, three-tissue response functions were estimated using the Dhollander algorithm and averaged across all participants to derive group-representative response functions (Dhollander et al., 2019). Preprocessed dMRI data were resampled to an isotropic voxel size of 1.25 mm prior to deconvolution. Fiber orientation distributions (FODs) were computed using single-shell three-tissue constrained spherical deconvolution (SS3T-CSD) (Dhollander and Connelly, 2016). Bias-field correction and global intensity normalization were performed across tissue compartments so that fixel metrics were comparable across participants (Dhollander et al., 2021; Raffelt et al., 2017).

**Fig. 1:**
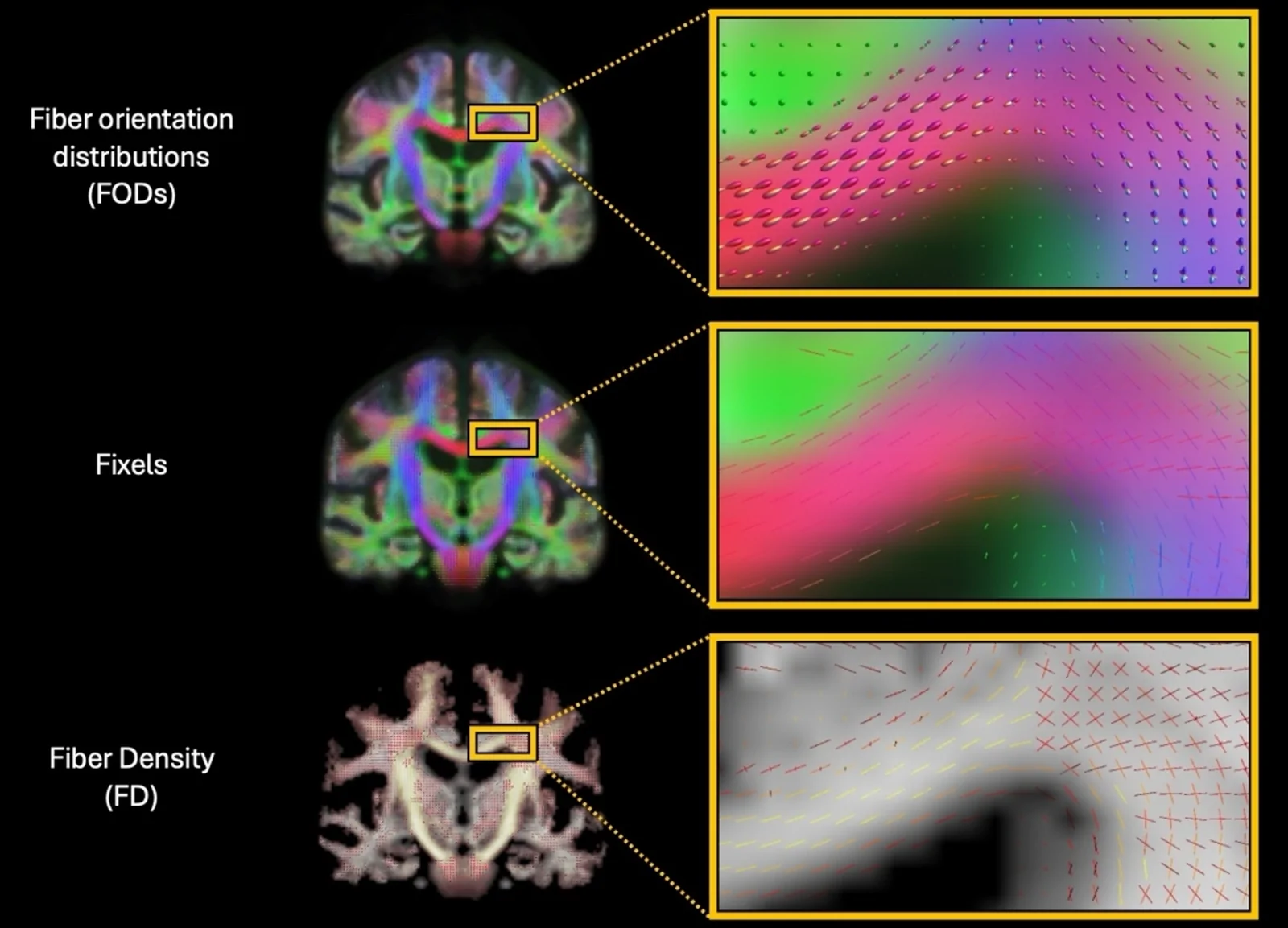
Computation of Fixel-wise Apparent Fiber Density from White Matter FODs. Top: White matter fiber orientation distributions (white matter FODs), estimated using single-shell three-tissue constrained spherical deconvolution (SS3T-CSD), serve as the basis for both the fixel grid and the apparent FD metric. FODs are visualized in a coronal slice overlaid on an FOD-based directionally encoded color (DEC) map (red = mediolateral, green = anteroposterior, blue = superoinferior). Middle: Fixels are derived by segmenting each voxel’s FOD into discrete lobes corresponding to distinct fiber populations, based on local angular maxima. In contrast to the regular voxel lattice, the spatial distribution and orientation of fixels are anatomically constrained by local white matter structure. Bottom: Apparent fiber density is calculated for each fixel as the integral of its corresponding FOD lobe. Voxel-wise FD (underlying grayscale intensities) reflects the sum of all fixel-wise FD values within each voxel

To enable spatial correspondence across subjects, a study-specific population FOD template was generated from a representative subset of 30 participants, including 15 individuals from each of the CU and MCI groups. Each participant’s FOD image was non-linearly registered to this template, and fixel-based metrics were derived in template space. Within each voxel, fixels were ordered by their group-averaged fiber density to define the primary (N1), secondary (N2), and tertiary (N3) fiber populations, providing a consistent anatomical definition of each population across participants. The ranking was derived once from fiber density and applied to all three metrics, so N1, N2, and N3 refer to the same fixels across FD, FC, and FDC. For each fixel metric, N1, N2, and N3 fiber populations within each voxel were projected onto separate voxel-wise maps using the fixel2voxel command in MRtrix3 (Grazioplene et al., 2018; Tournier et al., 2019). Voxels lacking a given population were treated as missing, rather than zero, for that population. Figure 1 shows representative grids from FOD, fixel, and fiber density maps.

**Fig. 2:**
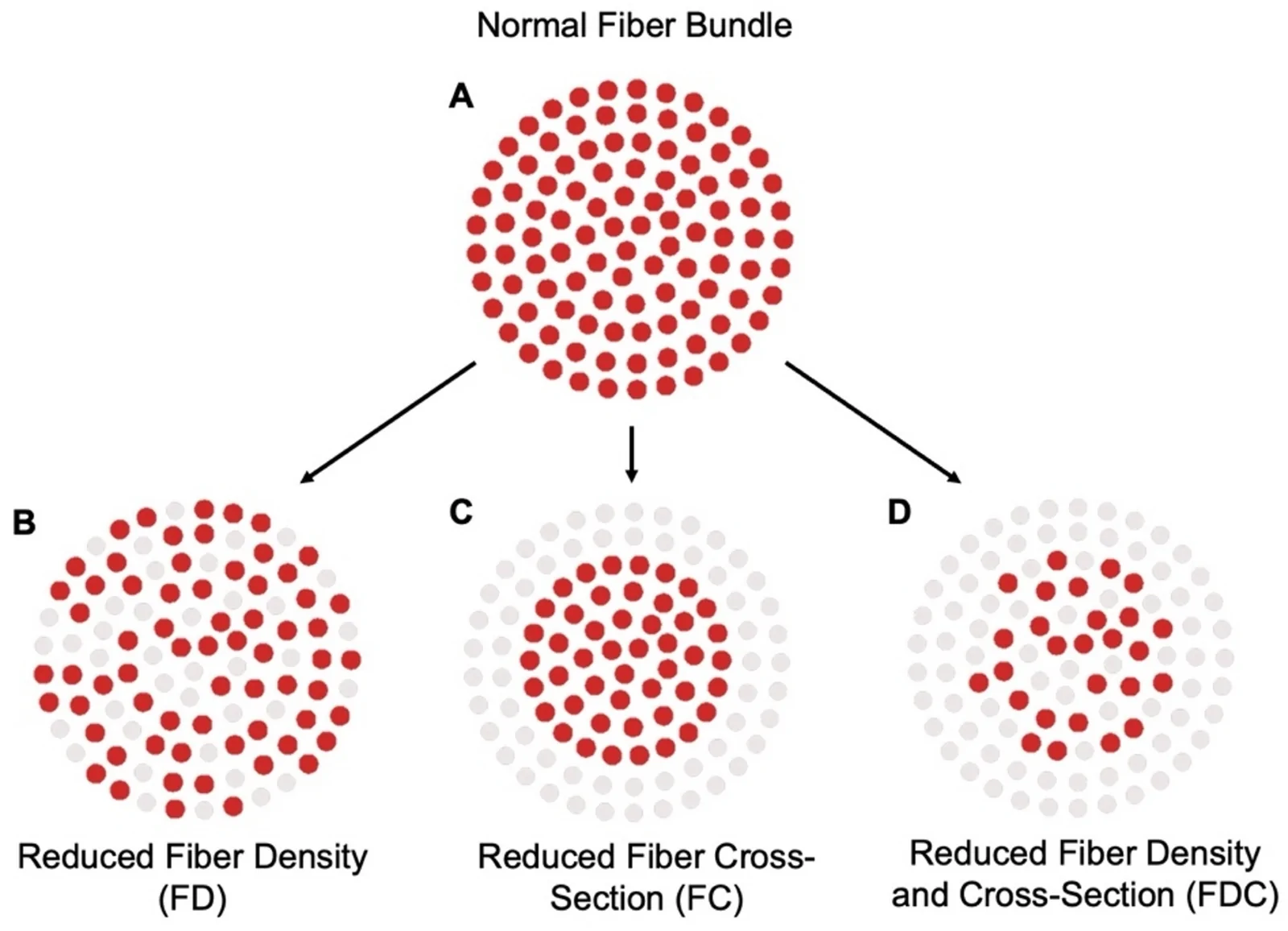
Schematic of the three fixel metrics, adapted from Raffelt et al. (2017). FBA characterizes white matter using three complementary metrics: fiber density (FD), a microstructural estimate of intra-axonal volume; fiber-bundle cross-section (FC), a macrostructural measure of morphological difference from the population template; and their product, fiber density and cross-section (FDC). **A** Normal fiber architecture. **B** Reduction in FD alone, consistent with axonal loss. **C** Reduction in FC alone, reflecting fiber-bundle contraction relative to the template. **D** Joint reduction in FD and FC, captured by FDC

The following fixel metrics were quantified and analyzed (illustrated in Fig. 2):

- Fiber Density (FD): A sub-voxel metric defined as the integral of the FOD along a given fixel direction and interpreted as an estimate of the intra-axonal volume of that fiber population. FD reflects the amplitude of a single FOD lobe and is influenced by the partitioning of the diffusion signal among the fiber populations that cross within a voxel. It is therefore best regarded as an apparent rather than an absolute measure of density.
- Fiber-Bundle Cross-Section (FC): A macrostructural metric quantifying the morphological difference in fiber-bundle diameter between an individual subject and the population template, computed as the deformation required in the plane perpendicular to the fixel orientation during spatial registration. All FC values reported here refer to the logarithmic transform of FC (log-FC). FC expresses morphological difference relative to the template rather than absolute fiber-bundle size, and differences in mean FC magnitude between fiber populations reflect this template-relative scaling rather than differences in absolute bundle caliber.
- Fiber Density and Cross-Section (FDC): A composite metric defined as the product of FD and FC that permits the simultaneous assessment of microstructural and macrostructural white matter properties. The product is taken with FC on its original scale rather than with log-FC. As FDC combines both components, opposing changes in FD and FC may partially offset within the composite measure.

### Stratification by crossing-fiber complexity

**Fig. 3:**
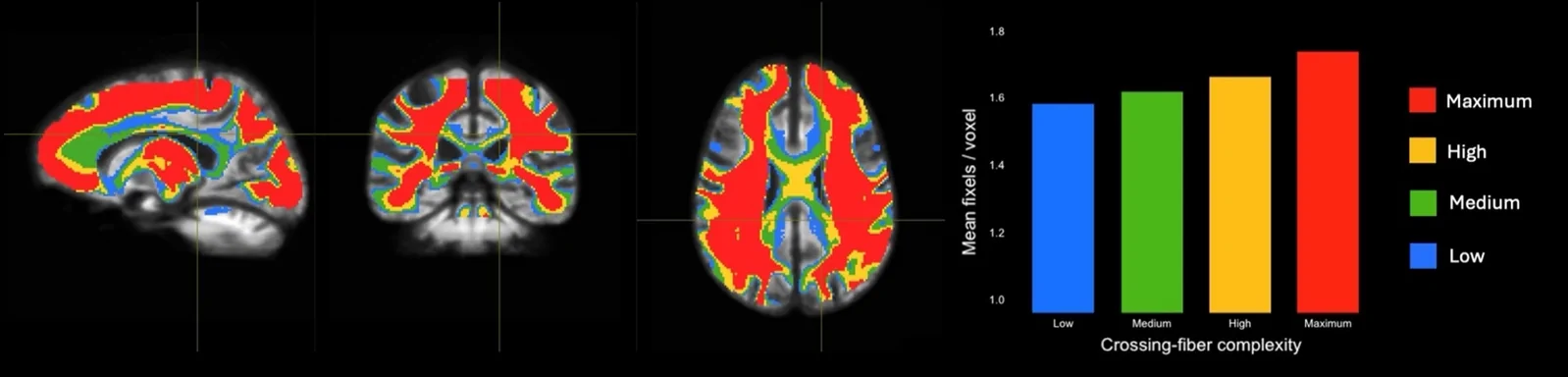
Crossing-fiber complexity strata. Left: sagittal, coronal, and axial views of the four white matter strata, defined by the number of distinct bundles traversing each voxel (Schilling et al., 2021): low (1 to 2 bundles, blue), medium (2 to 3, green), high (3 to 4, yellow), and maximum (4 or more, red). Voxels containing fewer than one bundle were excluded. Right: mean number of fixels per voxel within each stratum, computed from each participant’s own fiber orientation distributions and averaged across participants, showing that strata containing more bundles also contain more resolvable fiber populations

White matter voxels were stratified into four crossing-fiber complexity strata using a preexisting voxel-wise bundle overlap map (Schilling et al., 2021; https://www.nitrc.org/projects/bottlenecks), computed from 25 healthy Human Connectome Project participants rather than the present sample. In this map, each voxel intensity reflects the number of distinct white matter bundles traversing it. These counts were derived at the level of individual fixels and aggregated within each voxel, so the map is a voxel-wise representation of fixel-level bundle overlap. The map was non-linearly registered to the study-specific FOD template. Voxels containing fewer than one bundle were excluded to restrict analyses to regions with crossing-fiber architecture, and the remaining voxels were divided into four strata by bundle count: low (1 to 2), medium (2 to 3), high (3 to 4), and maximum (4 or more). Strata were defined by integer bundle count so that each corresponds to an interpretable number of crossing bundles. Because fixel metrics were averaged within regions rather than tested voxel-wise, the voxel-wise bundle-count map was reduced to this small number of regions, and each stratum was then represented by its mean bundle count, which was 1.5, 2.5, 3.5, and 6.5 bundles for the low, medium, high, and maximum strata respectively. This allowed crossing-fiber complexity to enter the statistical models as a continuous predictor rather than a categorical factor. The maximum stratum has no upper bound, so its mean is separated from the others by a larger interval than they are from one another. A binary mask was generated for each stratum in template space. For each participant, FD, FC, and FDC were averaged within each stratum separately for N1, N2, and N3, with voxels lacking a given population excluded from that average. Figure 3 shows representative cross-sections of the four strata in template space.

### Statistical analyses

#### General approach

All analyses were performed in R version 4.4.2, with mixed-effects models fitted using lmerTest (Kuznetsova et al., 2017) and marginal trends and contrasts obtained using emmeans (Lenth et al., 2026). Continuous predictors and fixel metrics were standardized across participants, except in descriptive summaries and figures, which retain raw metric units. Covariates comprised age, sex, education, intracranial volume, race/ethnicity, Framingham Stroke Risk Profile score excluding points for age, and *APOE*-ε4 carrier status, coded as positive (ε2/ε4, ε3/ε4, or ε4/ε4) or negative (ε2/ε2, ε2/ε3, or ε3/ε3). Cognitive status (CU or MCI) was included as a covariate when the outcome was a fixel metric and omitted when the outcome was a cognitive score, because clinical status is defined in part by cognitive performance.

Mixed-effects models were estimated by restricted maximum likelihood, with Type III F tests and Satterthwaite denominator degrees of freedom. Where a participant contributes more than one observation, as in the models stacked across fiber populations or complexity strata, a by-participant random intercept accounts for the resulting dependence. The complexity strata are non-overlapping regions of white matter, so the stacked structure represents multiple regional measurements per participant rather than repeated measurements of the same tissue. The Benjamini-Hochberg procedure (Benjamini and Hochberg, 1995) controlled the false discovery rate within each family of related tests, defined below for each analysis. Model specifications are summarized in Supplementary Table 1.

In the equations below, * i* indexes participants, * p* fiber populations, * s* complexity strata, * k* covariates, and $\:t\:$the interval in years since the baseline visit. $\:O\:$denotes the outcome of a given model, * Y* a standardized fixel metric, and $\:Z\:$a cognitive composite. * A* is standardized age and * X* the predictor of interest. $\:{\mathrm{N}2}_{p}\:$and $\:{\mathrm{N}3}_{p}$ are indicator variables for fiber population with N1 as the reference, and $\:{\stackrel{\prime }{B}}_{s}$ is the mean-centered empirical bundle count of stratum * s*. $\:{C}_{k}$ are the covariates listed above, excluding any that enter the model as the predictor of interest. $\:\beta\:\:$and $\:\gamma\:$ denote fixed-effect coefficients, * u* by-participant random effects, and $\:\epsilon\:$ residuals.

#### Group differences and global associations

Age associations with each global fixel metric and group differences in each fixel metric were estimated with linear regression, fitted separately for each of the nine fiber population by metric combinations (Eq. 1).

Linear model for cross-sectional associations within a single fiber population and metric.

1 $$\:{O}_{i}={\beta\:}_{0}+{\beta\:}_{1}{X}_{i}+\sum\:_{k}{\gamma\:}_{k}{C}_{ki}+{\epsilon\:}_{i}$$

The same form gave the adjusted group differences in cognitive performance, with a cognitive composite as outcome and cognitive status as predictor, and the baseline cognition associations, with a cognitive composite as outcome and the fixel metric as predictor. For the stratum-wise group differences the model was fitted separately within each complexity stratum, and the age by cognitive status interaction was tested by adding that interaction to the same form. The false discovery rate was controlled across the nine fiber population by metric combinations, and separately within each stratum for the stratified models.

#### Fiber population models

To test whether the age association differed across fiber populations, each metric was stacked across N1, N2 and N3 and modeled as a function of age, fiber population and their interaction, with a by-participant random intercept (Eq. 2).

Mixed-effects model for the interaction between age and fiber population.

2 $$\begin{aligned} \:{Y_{ip}} & = \beta {\:_0} + \beta {\:_1}{A_i} + \beta {\:_2}N{2_p} + \beta {\:_3}N{3_p} + \beta {\:_4}{A_i}N{2_p}\\ & + \beta {\:_5}{A_i}N{3_p} + \sum {{\:_k}} \gamma {\:_k}{C_{ki}} + {u_i} + \smallint {\:_{ip}} \end{aligned}$$

The interaction was evaluated with a likelihood-ratio test against the model omitting $\:{\beta\:}_{4}$ and $\:{\beta\:}_{5}$. Age slopes within each population and pairwise differences between them were obtained as estimated marginal trends, and the false discovery rate was then controlled across the nine population by metric slopes and across the nine pairwise contrasts as separate families.

#### Unified fiber population by crossing-fiber complexity models

To test whether the age and cognitive status associations varied with crossing-fiber complexity, and whether any such variation differed across fiber populations, each metric was stacked across the twelve fiber population by stratum combinations and modeled with a three-way specification, so that the population effect, the complexity effect and their joint effect are estimated within one model (Eq. 3). * X* denotes age for the analyses in Sect. 3.3 and cognitive status for those in Sect. 3.4.

Mixed-effects model for the interaction between age or cognitive status, fiber population, and crossing-fiber complexity.

3 $$\:\begin{array}{c}{Y}_{ips}={\beta\:}_{0}+{\beta\:}_{1}{X}_{i}+{\beta\:}_{2}{\mathrm{N}2}_{p}+{\beta\:}_{3}{\mathrm{N}3}_{p}+{\beta\:}_{4}{\stackrel{\prime }{B}}_{s}\\\:+{\beta\:}_{5}{X}_{i}{\mathrm{N}2}_{p}+{\beta\:}_{6}{X}_{i}{\mathrm{N}3}_{p}+{\beta\:}_{7}{X}_{i}{\stackrel{\prime }{B}}_{s}+{\beta\:}_{8}{\mathrm{N}2}_{p}{\stackrel{\prime }{B}}_{s}+{\beta\:}_{9}{\mathrm{N}3}_{p}{\stackrel{\prime }{B}}_{s}\\\:+{\beta\:}_{10}{X}_{i}{\mathrm{N}2}_{p}{\stackrel{\prime }{B}}_{s}+{\beta\:}_{11}{X}_{i}{\mathrm{N}3}_{p}{\stackrel{\prime }{B}}_{s}+\sum\:_{k}{\gamma\:}_{k}{C}_{ki}+{u}_{i}+{\epsilon\:}_{ips}\end{array}$$

Bundle count was centered on the mean of the four strata, so $\:{\beta\:}_{1}$, $\:{\beta\:}_{5}$ and $\:{\beta\:}_{6}$ are evaluated at the mean bundle count rather than at zero bundles. The complexity slope within each population was obtained as a linear combination of fixed effects, $\:{\beta\:}_{7}$ for N1 and $\:{\beta\:}_{7}+{\beta\:}_{10}$ or $\:{\beta\:}_{7}+{\beta\:}_{11}$ for N2 and N3. Slopes of * X* within each stratum were obtained as estimated marginal trends evaluated at $\:{\stackrel{\prime }{B}}_{s}$. Both sets of estimates are tested with Wald * z* statistics, since emmeans does not compute Satterthwaite degrees of freedom for models of this size. A sensitivity analysis replaced $\:{\stackrel{\prime }{B}}_{s}$ with the mean-centered ordinal rank of each stratum. The false discovery rate was controlled across the three metrics within each term of the model, across the nine population by metric complexity slopes, and across the nine population by metric slopes within each stratum, each as a separate family.

#### Cognition models

To test whether the association between a fixel metric and cognition differed across fiber populations or complexity strata, each composite was modeled on data stacked across cells, with every cell given its own intercept $\:{\alpha\:}_{g}$, metric slope $\:{\delta\:}_{g}$ and covariate coefficients (Eq. 4). Cells, indexed by * g*, are fiber populations in the population analysis and fiber population by stratum combinations in the complexity analysis.

Linear model for associations between fixel metrics and cognition within each cell.

4 $$\:{Z}_{ig}={\alpha\:}_{g}+{\delta\:}_{g}{Y}_{ig}+\sum\:_{k}{\gamma\:}_{kg}{C}_{ki}+{\epsilon\:}_{ig}$$

Each $\:{\delta\:}_{g}$ is therefore the slope estimated for that cell alone, and the tests below are differences between those slopes. Standard errors were clustered by participant using a bias-corrected sandwich estimator (Bell and McCaffrey, 2002; Liang and Zeger, 1986). Differences across populations were tested as the three pairwise contrasts among $\:{\delta\:}_{\mathrm{N}1}$, $\:{\delta\:}_{\mathrm{N}2}$ and $\:{\delta\:}_{\mathrm{N}3}$, with a joint Wald test of equality across all three. Moderation by complexity within a population was tested as the least-squares slope of that population’s four stratum estimates on the centered bundle count, which expresses the change in the metric to cognition slope per additional bundle, with a joint Wald test across the three populations.

Associations between baseline fixel metrics and longitudinal change in cognition were estimated with linear mixed-effects models including the interaction between each metric and the interval since baseline, with by-participant random intercepts and slopes (Eq. 5).

Linear mixed-effects model relating baseline fixel metrics to longitudinal change in cognition.

5 $$\begin{aligned} \:{Z_{it}} & = \beta {\:_0} + \beta {\:_1}{Y_i} + \beta {\:_2}{t_{it}} + \beta {\:_3}{Y_i}{t_{it}}\\ & + \sum {{\:_k}} \gamma {\:_k}{C_{ki}} + {u_{0i}} + {u_{1i}}{t_{it}} + \smallint {\:_{it}} \end{aligned}$$

The false discovery rate was controlled across the nine population contrasts within each cognitive domain, across the nine complexity slopes within each domain, across the six joint Wald tests in each of the two moderation analyses, and across the nine population by metric combinations for each cognitive measure in the baseline and longitudinal models, each as a separate family.

The memory and executive function composites, each derived from multiple standardized tests using a previously validated approach (Kresge et al., 2018), were designated the primary cognitive outcomes, as aggregation across indicators attenuates test-specific measurement error. Individual measures of language, information processing speed and visuospatial ability were analyzed identically and are reported in the supplementary materials.

## Results

### Participants and white matter architecture

The analytic sample comprised 295 older adults from VMAP. Full demographic and clinical characteristics are reported in Table 1. CU and MCI participants were comparable in age, sex, intracranial volume, and race/ethnicity, whereas participants with MCI had fewer years of education, higher Framingham Stroke Risk Profile scores, a greater proportion of *APOE*-ε4 carriers, and fewer study visits over a shorter follow-up period. All cognitive scores were worse in the MCI group after adjustment for covariates.

The mean number of fixels per voxel increased with greater bundle count, from 1.58 in the low stratum to 1.74 in the maximum stratum (Fig. 3), and all pairwise differences between strata were significant in paired t tests (all p_FDR_ < 0.001), indicating that regions containing more distinct bundles also contained more resolvable fiber populations. Mean FD, FC, and FDC within each fiber population and stratum are reported in Supplementary Table 2. These values did not vary monotonically, reflecting that the strata comprise anatomically distinct white matter regions that differ in their regional composition in addition to their crossing-fiber complexity.

**Table 1:** Vanderbilt Memory and Aging Project (VMAP) cohort information

	Total	CU	MCI	*p*-value (CU vs. MCI)
Number of participants	295	166	129	–
Number of sessions	1193	840	353	–
Number of visits	3.05 ± 1.70	3.29 ± 1.71	2.48 ± 1.55	< 0.001
Longitudinal follow-up (years)	4.58 ± 2.69	4.81 ± 2.71	3.86 ± 2.49	< 0.001
Age at baseline	72.86 ± 7.37	72.51 ± 7.23	73.32 ± 7.55	0.355
Sex (% Female)	126 (42.71)	69 (41.57)	57 (44.19)	0.739
Race (% NHW)	256 (86.78)	145 (87.35)	111 (86.05)	0.877
Education (years)	15.81 ± 2.69	16.40 ± 2.49	15.06 ± 2.76	< 0.001
*APOE*-ε4 positivity (% positive)	106 (35.93)	50 (30.12)	56 (43.41)	0.019
FSRP _subtracting age points_	6.31 ± 3.12	5.96 ± 2.97	6.75 ± 3.28	0.034
Intracranial volume (cm^3^)	1379.5 ± 143.9	1381.2 ± 139.8	1377.2 ± 149.6	0.818
Composite memory score	−0.02 ± 0.98	0.55 ± 0.73	−0.75 ± 0.75	< 0.001
Composite executive function score	−0.01 ± 0.93	0.43 ± 0.61	−0.59 ± 0.95	< 0.001
Language				
Animal naming test	18.77 ± 5.53	20.83 ± 4.89	16.13 ± 5.20	< 0.001
Boston naming test	26.75 ± 3.23	27.87 ± 2.02	25.32 ± 3.87	< 0.001
Information processing speed				
DKEFS Number Sequencing	42.89 ± 21.18	36.04 ± 12.67	51.71 ± 26.16	< 0.001
WAIS-IV Coding	52.47 ± 13.04	57.26 ± 11.77	46.32 ± 12.00	< 0.001
Visuospatial skills				
Hooper visual organization test	24.38 ± 3.22	25.30 ± 2.48	23.19 ± 3.66	< 0.001

Continuous variables were compared with independent-samples t tests and categorical variables with chi-square tests

Values are unadjusted means ± standard deviations or counts (percentages)

*NHW* non-hispanic white. p-values for cognitive measures are from covariate-adjusted models and are reported without correction for multiple comparisons

### Age associations with fixel metrics across fiber populations

Associations between age and each global fixel metric are reported in Table 2 (Eq. 1). FD showed a single significant association, a positive relationship in N2 (β = 0.20, pFDR = 0.003). FC decreased with age in N1, N2, and N3, with the largest associations in N2 (β = −0.11, pFDR < 0.001) and N3 (β = −0.10, pFDR < 0.001) and a smaller association in N1 (β = −0.04, pFDR = 0.040). FDC decreased with age in all three populations and reached significance in N3 (β = −0.07, pFDR = 0.020). Because the age by population interaction was not significant for FDC, as reported below, this association is interpreted as comparable across N1, N2, and N3 rather than specific to N3.

**Table 2:** Age associations with global fixel metrics

Metric	Population	β	SE	CI_Low_	CI_High_	t	*p*	*p*_FDR_
FD	N1	−0.003	0.060	−0.121	0.116	−0.042	0.966	0.966
FD	N2	0.204	0.060	0.085	0.323	3.373	**0.001**	**0.003**
FD	N3	0.044	0.061	−0.076	0.165	0.725	0.469	0.528
FC	N1	−0.036	0.016	−0.066	−0.005	−2.298	**0.022**	**0.040**
FC	N2	−0.108	0.018	−0.142	−0.073	−6.079	**< 0.001**	**< 0.001**
FC	N3	−0.100	0.015	−0.130	−0.069	−6.511	**< 0.001**	**< 0.001**
FDC	N1	−0.048	0.034	−0.114	0.018	−1.420	0.157	0.201
FDC	N2	−0.042	0.029	−0.098	0.015	−1.447	0.149	0.201
FDC	N3	−0.074	0.028	−0.129	−0.018	−2.628	**0.009**	**0.020**

β is the standardized age slope, adjusted for the covariates in Sect. 2.6.1, with 285 degrees of freedom. CI is the 95% confidence interval. pFDR is the Benjamini-Hochberg adjustment across the nine fiber population by metric combinations. Bold values denote statistical significance (p < 0.05).

To test whether these associations differed across N1, N2, and N3, each metric was modeled as a function of age, fiber population, and their interaction, with a by-participant random intercept (Eq. 2). The interaction was significant for FC and FD but not FDC, and the age slopes estimated within these models are reported in Supplementary Table 3, where the smaller N1 FC association falls just above the correction threshold (pFDR = 0.058). For FD, the age slope was more positive in N2 than in either N1 (pFDR < 0.001) or N3 (pFDR = 0.020), so this positive association was specific to N2 rather than shared across the non-dominant populations. Pairwise contrasts are reported in Table 3. For FC, the age-related decrease was steeper in N2 and N3 than in N1 (both pFDR < 0.001), with no difference between N2 and N3 (pFDR = 0.552). The absence of a population interaction for FDC, despite interactions in both of its components, follows from the opposing directions of the FD and FC age associations and is addressed in the Discussion. Age did not interact with cognitive status for any fixel metric or fiber population (all pFDR = 0.78; Supplementary Table 4).

**Table 3:** Differences in the age slope across fiber populations

Metric	Contrast	Estimate	CI_Low_	CI_High_	t	*p*	*p*_FDR_	Interaction *p*
FD	N1 - N2	−0.216	−0.320	−0.112	−4.090	**< 0.001**	**< 0.001**	**< 0.001**
FD	N1 - N3	−0.077	−0.181	0.027	−1.460	0.145	0.261	
FD	N2 - N3	0.139	0.035	0.243	2.631	**0.009**	**0.020**	
FC	N1 - N2	0.072	0.056	0.089	8.691	**< 0.001**	**< 0.001**	**< 0.001**
FC	N1 - N3	0.066	0.050	0.083	8.002	**< 0.001**	**< 0.001**	
FC	N2 - N3	−0.006	−0.022	0.011	−0.689	0.491	0.552	
FDC	N1 - N2	−0.005	−0.046	0.036	−0.260	0.795	0.795	0.389
FDC	N1 - N3	0.022	−0.019	0.063	1.035	0.301	0.387	
FDC	N2 - N3	0.027	−0.014	0.068	1.295	0.196	0.294	

Estimate is the difference in the standardized age slope between populations. Interaction p is the likelihood-ratio test for the age-by-population interaction. Contrast p-values are unadjusted, and pFDR is the Benjamini-Hochberg adjustment across all nine contrasts. CI is the 95% confidence interval. Contrasts are estimated with 584 degrees of freedom. Bold values denote statistical significance (p < 0.05).

### Moderation of age associations by crossing-fiber complexity

To assess whether these age associations varied with local crossing-fiber complexity, each metric was modeled as a function of age, fiber population, the bundle-count value of each complexity stratum, and all interactions among the three, with a by-participant random intercept (Eq. 3). Complexity was represented by the mean-centered empirical bundle count of each stratum, so that lower-order terms are evaluated at the mean bundle count, and results were consistent when strata were represented by ordinal rank (Supplementary Table 5). The hierarchy of interaction tests is reported in Table 4. The age by population interaction reproduced the pattern obtained on the global maps in Sect. 3.2. The age by complexity interaction was significant for all three metrics (all pFDR < 0.001), and the three-way interaction was significant for FD (F = 6.06, pFDR = 0.004) and FC (F = 12.55, pFDR < 0.001) but not FDC (F = 0.17, pFDR = 0.845). Complexity therefore modified the age association for every metric, and did so to a different degree across fiber populations for FD and FC.

**Table 4:** Age interactions with fiber population and crossing-fiber complexity

Metric	Term	F	*p*	*p*_FDR_
FD	Age	3.734	0.054	0.067
FD	Age ✕ Population	49.558	**< 0.001**	**< 0.001**
FD	Age ✕ Complexity	63.212	**< 0.001**	**< 0.001**
FD	Age ✕ Population ✕ Complexity	6.059	**0.002**	**0.004**
FC	Age	31.125	**< 0.001**	**< 0.001**
FC	Age ✕ Population	82.766	**< 0.001**	**< 0.001**
FC	Age ✕ Complexity	20.120	**< 0.001**	**< 0.001**
FC	Age ✕ Population ✕ Complexity	12.555	**< 0.001**	**< 0.001**
FDC	Age	3.374	0.067	0.067
FDC	Age ✕ Population	0.747	0.474	0.474
FDC	Age ✕ Complexity	14.293	**< 0.001**	**< 0.001**
FDC	Age ✕ Population ✕ Complexity	0.169	0.845	0.845

F tests are Type III with Satterthwaite denominator degrees of freedom. Crossing-fiber complexity is the mean-centered empirical bundle count of each stratum, so the lower-order terms are evaluated at the mean bundle count. The age term represents the age effect averaged across the three fiber populations. pFDR is the Benjamini-Hochberg adjustment across metrics within each term. Numerator degrees of freedom are 1 for the lower-order and complexity terms and 2 for the population and three-way terms. Denominator degrees of freedom are 285 for the lower-order term and 3224 for the interaction terms. Bold values denote statistical significance (p < 0.05).

Complexity slopes within each fiber population are reported in Supplementary Table 6, expressed as the change in the standardized age slope per additional bundle, and age slopes within each stratum in Supplementary Table 7. For FC, the complexity effect was confined to N1 (− 0.017, pFDR < 0.001) and was absent in N2 (pFDR = 0.631) and N3 (pFDR = 0.610). FC accordingly declined with age at a nearly constant rate across the four strata in N2 (− 0.115 to − 0.121) and N3 (− 0.108 to − 0.116), with all eight estimates significant at pFDR < 0.001, whereas in N1 the association was absent in the low stratum (− 0.005, pFDR = 0.754) and steepest in the maximum stratum (− 0.090, pFDR < 0.001).

For FD, the complexity effect was present in all three populations and largest in N1 (0.071, pFDR < 0.001). The age association with N1 FD reversed in sign across the strata, from − 0.182 in the low stratum (pFDR = 0.003) to 0.172 in the maximum stratum (pFDR = 0.005), while N2 FD was positive at every stratum and increased from 0.150 to 0.293. FDC slopes were negative at every stratum, attenuated toward zero as complexity increased, and did not differ across fiber populations. The age-related decline in non-dominant FC was the only age association that did not vary with crossing-fiber complexity.

### Fixel-metric differences by cognitive status

Differences in each global fixel metric between CU and MCI participants are reported in Table 5. FC was lower in the MCI group in N2 (β = −0.11, pFDR = 0.007) and N3 (β = −0.10, pFDR = 0.007), and FD and FDC were lower in this group in N1 (β = −0.34, pFDR = 0.013, and β = −0.19, pFDR = 0.013). FD was higher in the MCI group in N3, although this difference did not survive correction (β = 0.26, pFDR = 0.065). No other differences survived correction.

**Table 5:** Differences in global fixel metrics between diagnostic groups

Metric	Population	β	SE	CI_Low_	CI_High_	t	*p*	*p*_FDR_
FD	N1	−0.340	0.122	−0.580	−0.100	−2.786	**0.006**	**0.013**
FD	N2	0.154	0.122	−0.086	0.394	1.263	0.208	0.267
FD	N3	0.260	0.124	0.017	0.504	2.107	**0.036**	0.065
FC	N1	−0.062	0.031	−0.123	0.0002	−1.961	0.051	0.076
FC	N2	−0.114	0.036	−0.184	−0.044	−3.193	**0.002**	**0.007**
FC	N3	−0.105	0.031	−0.166	−0.044	−3.397	**0.001**	**0.007**
FDC	N1	−0.191	0.068	−0.325	−0.058	−2.826	**0.005**	**0.013**
FDC	N2	−0.069	0.058	−0.183	0.046	−1.180	0.239	0.269
FDC	N3	−0.011	0.057	−0.122	0.100	−0.199	0.843	0.843

β is the standardized difference for the MCI group relative to the CU group. Negative values indicate lower metric values in MCI. CI is the 95% confidence interval. Models are adjusted for the covariates in Sect. 2.6.1 with 285 degrees of freedom. pFDR is the Benjamini-Hochberg adjustment across the nine fiber population by metric combinations. Bold values denote statistical significance (p < 0.05).

To test whether these differences varied across fiber populations or complexity strata, each metric was modeled as a function of cognitive status, fiber population, the bundle-count value of each stratum, and all interactions among the three, as in Sect. 3.3 (Eq. 3). Averaged across fiber populations at the mean bundle count, the group difference was significant for FC only (F = 8.39, pFDR = 0.012). The status by population interaction was significant for all three metrics (all pFDR < 0.001). The status by complexity interaction was significant for FC (F = 9.65, pFDR = 0.006) and was not significant for FD (pFDR = 0.317) or FDC (pFDR = 0.233). The three-way interaction was not significant for any metric.

**Table 6:** Cognitive status interactions with fiber population and crossing-fiber complexity

Metric	Term	F	*p*	*p*_FDR_
FD	Cognitive status	0.144	0.705	0.705
FD	Cognitive status ✕ Population	88.114	**< 0.001**	**< 0.001**
FD	Cognitive status ✕ Complexity	1.003	0.317	0.317
FD	Cognitive status ✕ Population ✕ Complexity	1.081	0.339	0.509
FC	Cognitive status	8.386	**0.004**	**0.012**
FC	Cognitive status ✕ Population	14.960	**< 0.001**	**< 0.001**
FC	Cognitive status ✕ Complexity	9.646	**0.002**	**0.006**
FC	Cognitive status ✕ Population ✕ Complexity	2.502	0.082	0.246
FDC	Cognitive status	1.990	0.159	0.239
FDC	Cognitive status ✕ Population	29.473	**< 0.001**	**< 0.001**
FDC	Cognitive status ✕ Complexity	2.020	0.155	0.233
FDC	Cognitive status ✕ Population ✕ Complexity	0.176	0.839	0.839

F tests are Type III with Satterthwaite denominator degrees of freedom. Crossing-fiber complexity is the mean-centered empirical bundle count of each stratum, so the lower-order terms are evaluated at the mean bundle count. The cognitive status term represents the group difference averaged across the three fiber populations. pFDR is the Benjamini-Hochberg adjustment across metrics within each term. Cognitive status contrasts the MCI group with the CU group. Numerator degrees of freedom are 1 for the lower-order and complexity terms and 2 for the population and three-way terms. Denominator degrees of freedom are 285 for the lower-order term and 3224 for the interaction terms. Bold values denote statistical significance (p < 0.05).

**Fig. 4:**
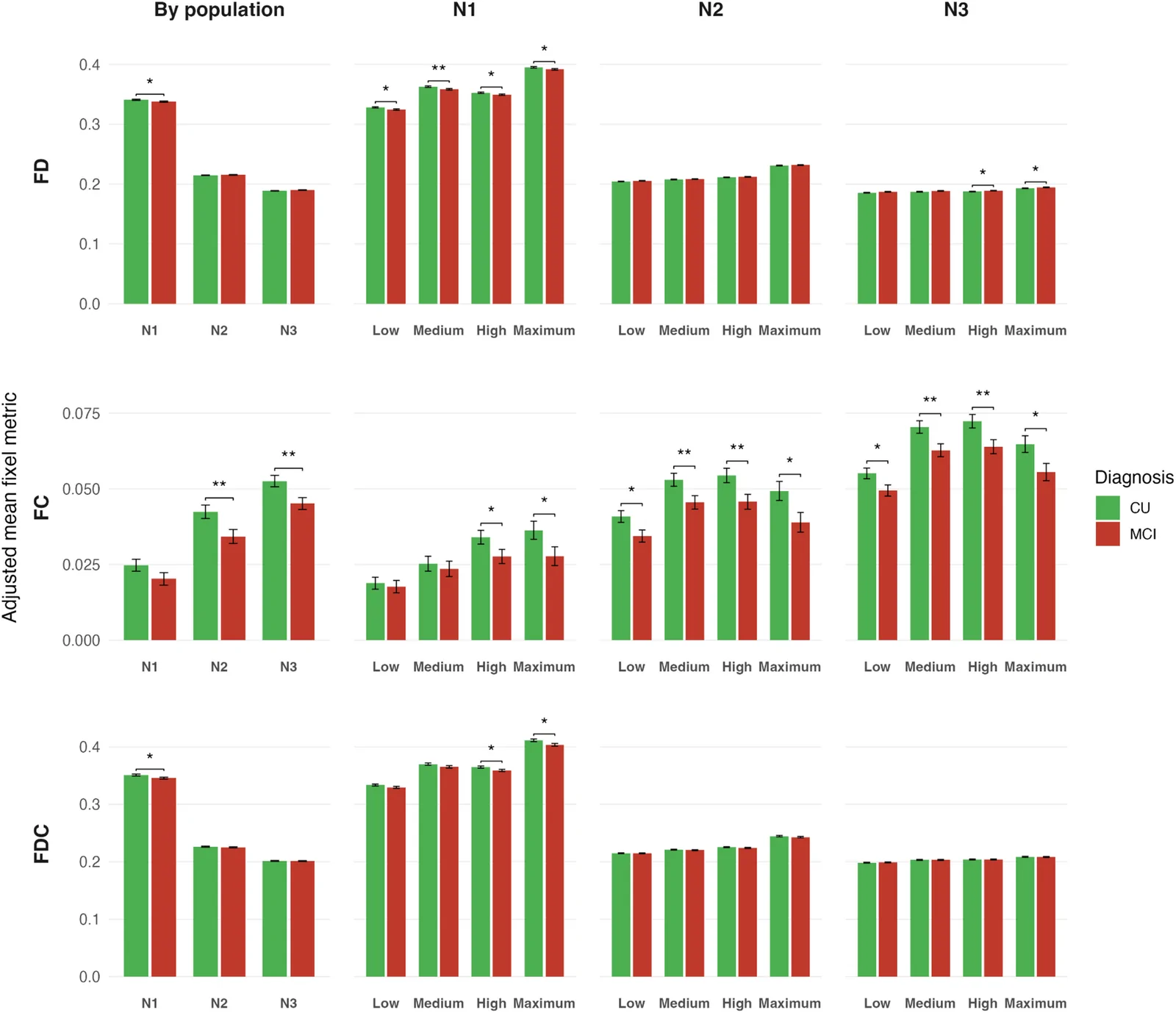
Covariate-adjusted fixel metrics by diagnostic group across fiber populations and crossing-fiber complexity strata. Estimated marginal means with standard errors for FD (top), FC (middle), and FDC (bottom) in cognitively unimpaired (CU) and mild cognitive impairment (MCI) participants (*N* = 295), in raw metric units and adjusted for the covariates listed in Sect. 2.6.1. Left column: fiber populations on the global maps. Remaining columns: each fiber population across the four complexity strata. Brackets with asterisks denote the adjusted group difference after false discovery rate correction, from Table 5 (global) and Supplementary Table 8 (strata). *pFDR < 0.05, **pFDR < 0.01. Vertical scales are free across metrics. Strata comprise anatomically distinct white matter regions and are not directly comparable

The same comparisons estimated within each complexity stratum are reported in Supplementary Tables 8 and are shown in Fig. 4. Lower N2 and N3 FC in the MCI group was present at each of the four strata, with all eight estimates surviving correction, and increased in magnitude with complexity, from − 0.09 to − 0.14 in N2 and from − 0.08 to − 0.13 in N3. The gradient did not differ across populations, and in N1, where the overall group difference was smaller, lower FC reached significance only in the high and maximum strata. Lower N1 FD was significant at every stratum and lower N1 FDC at the high and maximum strata, although neither difference varied significantly with complexity (Table 6). The stratum-wise estimates and the significance markers in Fig. 4 describe the group difference within each stratum rather than its change across strata. The dependence of the FC group difference on crossing-fiber complexity contrasts with the age-related decline in non-dominant FC, which did not vary across strata (Sect. 3.3).

### Associations between fixel metrics and cognition

Associations between baseline global fixel metrics and baseline cognition are reported in Supplementary Table 9. For the executive function composite, FC was positively associated with performance in N2 (β = 0.53, 95% CI [0.22, 0.84], t = 3.35, pFDR = 0.008) and N3 (β = 0.57, 95% CI [0.21, 0.93], t = 3.10, pFDR = 0.010). No association with the memory composite survived correction. A comparable pattern of positive associations between FC and performance was present for several individual neuropsychological measures.

To test whether these associations were specific to particular fiber populations or complexity strata, each composite was modeled as a function of the fixel metric, fiber population or the bundle-count value of each stratum, and their interaction, with each cell given its own slope and covariate adjustment and with standard errors clustered by participant (Eq. 4; Supplementary Tables 10 and 11). The association did not differ across fiber populations for any metric or domain (omnibus pFDR ≥ 0.15). The significant associations in N2 and N3 FC should therefore not be read as evidence that the association is absent in N1, in which the estimate was positive but did not reach significance (β = 0.34, pFDR = 0.157). Crossing-fiber complexity did not moderate the FC association in either domain (pFDR = 0.250 for memory and 0.341 for executive function). For FD, complexity moderated the executive function association (F = 4.01, pFDR = 0.049), although no individual population slope survived correction.

Associations between baseline fixel metrics and longitudinal change in cognition were estimated with linear mixed-effects models that included the interaction between each baseline metric and the interval from baseline (Eq. 5). No metric-by-interval interaction survived correction for the memory or executive function composites or for any individual measure (Supplementary Table 12).

## Discussion

This study used single-shell three-tissue constrained spherical deconvolution to compute fixel-based metrics in an aging cohort, decomposed each white matter voxel into primary (N1), secondary (N2), and tertiary (N3) fiber populations, and stratified the white matter by crossing-fiber complexity. FC declined with age most steeply in the non-dominant fiber populations, and this reduction both distinguished participants with MCI and tracked executive function. Fiber-population identity, rather than crossing-fiber complexity, organized the age associations, whereas the reduction in FC in MCI was larger in regions of higher complexity. FD, by contrast, showed a positive age association in N2, and the age associations of both FD and FDC became more positive as crossing-fiber complexity increased, a pattern more consistent with the way density is estimated in multi-fiber voxels than with fiber loss. The results identify the morphological integrity of non-dominant fiber populations, indexed by FC, as a sensitive marker of white matter aging, and indicate that the density-based metrics require careful interpretation when fiber populations are examined separately.

The central finding is that age-related reductions in FC are graded by fiber-population identity, with steeper decline in N2 and N3 than in N1 and no difference between N2 and N3. Because FC is a macrostructural measure derived from the deformation required to align a bundle to the population template (Raffelt et al., 2017), this pattern reflects greater morphological atrophy of non-dominant bundles with advancing age. Non-dominant fiber populations are likely to include later-developing, more thinly myelinated association fibers, which myelinate last and are more vulnerable to metabolic stress and age-related change (Bender and Raz, 2015; Kochunov et al., 2009; Marner et al., 2003; Peters, 2002). This interpretation aligns with the retrogenesis account of white matter aging, in which pathways that mature last show the earliest decline (Brickman et al., 2012; Fjell et al., 2014; Reisberg et al., 1999), and with the disconnection account of cognitive aging, in which loss of structural integrity in distributed pathways impairs network communication (Andrews-Hanna et al., 2007; O’Sullivan et al., 2001). The findings extend prior work from this cohort and others implicating association and limbic tracts in cognitive aging (Archer et al., 2020; Bender et al., 2016, 2020; Peter et al., 2025; Stone et al., 2021) by localizing the effect to the non-dominant fiber populations within those regions.

The non-dominant FC that was most sensitive to age also distinguished cognitive status. FC in N2 and N3 was lower in participants with MCI, and higher non-dominant FC was associated with better executive function. The association with executive function agrees with the dependence of executive processes on distributed frontal and association white matter networks (Gunning-Dixon and Raz, 2000; Kennedy and Raz, 2009). A comparable pattern of associations between FC and cognition was present for several individual measures spanning language, processing speed, and visuospatial ability. The association did not differ significantly across fiber populations, so although it reached significance in N2 and N3 and not in N1, the populations cannot be distinguished on this measure. The group difference in non-dominant FC was present at every crossing-fiber complexity stratum and increased in magnitude as complexity increased, so unlike the age association it did depend on local fiber complexity. In contrast, baseline fixel metrics did not predict longitudinal change in memory or executive function after adjustment for multiple tests. One interpretation is that cross-sectional FC may index the white matter integrity that accompanies current cognitive status more than the trajectory of future change, which is likely to be driven by the progressive accumulation of pathology not captured at a single time point (Jack and Holtzman, 2013). The longitudinal result is therefore best read as evidence that a single baseline measurement of these metrics is not sufficient to predict cognitive decline in this sample, rather than as evidence against a relationship between white matter integrity and decline.

FD and FDC behaved differently from FC. FD increased with age in N2, a direction opposite to that expected if aging reduced fiber density. This pattern is more consistent with the way FD is estimated than with a biological increase in axons. FD is the integral of a single lobe of the fiber orientation distribution, and within a voxel the total diffusion signal is apportioned among the resolved lobes (Raffelt et al., 2012, 2017). Age-related broadening of the fiber orientation distribution and, at the diffusion weighting used here, contributions from extra-axonal water can inflate the apparent density assigned to the non-dominant lobes without a corresponding change in axonal content (Dhollander et al., 2021; Raffelt et al., 2012). The stratified models support this account directly. The age association with N1 FD reversed from negative in the least complex stratum to positive in the most complex, and the associations in N2 and N3 became more positive as complexity increased, a dependence on the number of lobes sharing the voxel signal that apportionment predicts and fiber loss does not. Prior fixel-based studies of aging and AD have reported reductions in fiber density consistent with fiber loss, including within the fornix, cingulum, and SLF (Bhattarai et al., 2023; Billaud et al., 2024; Mito et al., 2018) and within striatal and thalamic pathways in CU older adults (Tinney et al., 2024). These studies examine fixels matched in anatomical correspondence across participants, summarizing metrics over fiber populations. In the present study, populations are defined by their density rank within a voxel rather than by anatomical identity. Our findings therefore cannot be compared directly with the population-averaged reductions reported for specific tracts or regions.

The divergence between FD and FC also accounts for the behavior of their composite. Because the age effect on FD was positive and the age effect on FC was negative, the two components offset one another in FDC, which showed no difference in its age association across fiber populations and no difference in how that association varied with crossing-fiber complexity. This nullification shows that a composite metric can obscure opposing microstructural and morphological effects and supports analyzing FD and FC separately (Raffelt et al., 2017). More broadly, the behavior of FD in the non-dominant populations illustrates the limited specificity of density estimates in multi-fiber voxels (Dhollander et al., 2021), a pattern that is apparent only when fiber populations are resolved. Comparisons of FD between fiber populations should therefore be interpreted with attention to how the within-voxel signal is apportioned. FC is derived from the macroscopic registration warp and does not involve apportioning a shared within-voxel signal (Raffelt et al., 2017), and its age associations can accordingly be interpreted directly in terms of bundle morphology.

The stratification by crossing-fiber complexity was designed to test whether the vulnerability of the non-dominant fiber populations depended on local fiber architecture. The age association of non-dominant FC did not vary across the complexity strata, changing by less than 0.01 in both N2 and N3 from the least to the most complex stratum. Complexity modified the age association for all three metrics, but for FC the effect was confined to the primary population, where the association was absent in the least complex stratum and steepest in the most complex. For FD the complexity dependence was present in every population, and for FDC it did not differ across populations, in both cases in the direction attributable to the apportionment effect described above. Fiber-population identity therefore appears to be a more salient organizing dimension of white matter aging in this sample than the degree of local crossing, at least for the age associations. The reduction in FC in MCI was larger in more complex regions, so the clinical contrast was sensitive to local architecture in a way the age association was not. This conclusion is qualified by the nature of the strata, which are derived from a population atlas of bundle counts (Schilling et al., 2021) and comprise anatomically distinct white matter regions. Crossing-fiber complexity is consequently confounded with tract identity and location, and the mean fixel-metric values did not vary monotonically across strata. Because the stratification does not isolate crossing complexity from regional anatomy, we do not draw strong architectural conclusions in either direction.

This study has several strengths. It applied single-shell three-tissue constrained spherical deconvolution, a reconstruction developed for single-shell acquisitions (Dhollander and Connelly, 2016), in a large and well-characterized aging cohort. By resolving primary, secondary, and tertiary fiber populations, the analysis characterized white matter aging at a level of fiber-specific detail that voxel-averaged approaches cannot provide (Raffelt et al., 2015). The crossing-fiber stratification, derived from an existing atlas rather than from tractography of the study sample, provides a framework that can be applied to other single-shell cohorts with standardized diffusion protocols. Several limitations should also be considered. The diffusion data were acquired at a single, relatively low diffusion weighting, which limits the specificity of FD and contributes to the apportionment behavior described above (Dhollander et al., 2021; Raffelt et al., 2012). Although the processing pipeline used here has been validated for single-shell data (Luo et al., 2021; Nguyen et al., 2021; Petersen et al., 2022), higher b-value or multi-shell acquisitions would improve the interpretability of density estimates. Because diffusion imaging was obtained only at baseline, all fixel metrics are cross-sectional, and within-person change in white matter microstructure could not be examined. Finally, metrics were averaged within global and complexity-stratified regions rather than resolved voxel-wise or by tract, which reduced the burden of multiple comparisons but precludes precise anatomical localization.

These findings suggest several extensions. Assigning N1, N2, and N3 to specific named pathways across regions would require tractography and represents a natural next step. Higher diffusion weighting or multi-shell acquisitions would sharpen the estimation of FD in multi-fiber voxels and allow the density and cross-section components to be interpreted with greater confidence. Longitudinal diffusion imaging would permit fiber-specific microstructure to be tracked within individuals and would test directly whether non-dominant fiber degeneration precedes cognitive decline. Finally, integrating fixel metrics with molecular markers of AD pathology and with spatially resolved analyses of fiber architecture would further clarify the microstructural basis of cognitive aging.

## Conclusion

This study shows that FBA provides a sensitive and fiber-specific approach for characterizing white matter changes in aging and MCI. By resolving FD and FC across primary, secondary, and tertiary fiber populations, we identified patterns of age- and disease-related difference that voxel-averaged diffusion methods cannot recover, and we showed that FC in non-dominant fiber populations is the most interpretable of these signals. Non-dominant FC declined with age, was reduced in MCI, and was associated with cognitive performance, whereas the complexity-dependent behavior of FD reflects the limitations of density estimates in crossing regions. The stability of the age-related decline in non-dominant FC across crossing-fiber complexity indicates that fiber-population identity is a more salient determinant of age-related white matter vulnerability than local anatomical convergence, although the reduction in MCI was larger in more complex regions. By resolving fiber-specific white matter microstructure, the framework applied here can be extended to existing single-shell cohorts and to longitudinal and multimodal studies of white matter vulnerability in neurodegeneration.

## Supplementary Information

Below is the link to the electronic supplementary material.

Supplementary Material 1

## Data Availability

Data from the VMAP cohort can be accessed freely following data use approval (www.vmacdata.org). The source code for the analysis conducted in this study is available on request.
